# Supplementary material for: The causal relationship between the human gut microbiota and pyogenic arthritis: a Mendelian randomization study
Source: Front Cell Infect Microbiol. 2024 Nov 26;14:1452480. doi: 10.3389/fcimb.2024.1452480 (PMC11629706; doi:10.3389/fcimb.2024.1452480)
Supplement: Supplementary file 1 [file DataSheet1.zip › mendelian test/ebi-a-GCST90027600.csv_forest.pdf]

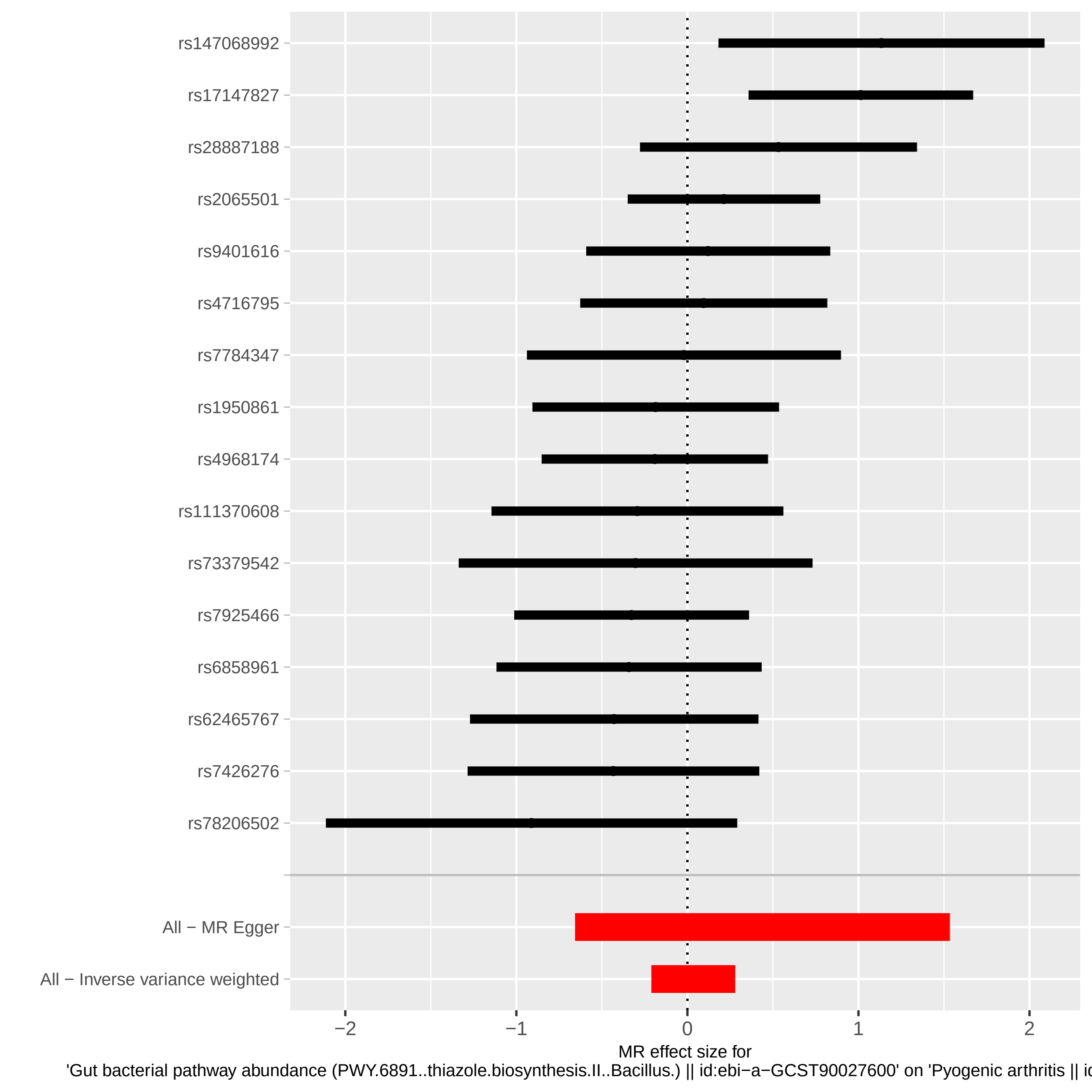
rs147068992
rs17147827
rs28887188
rs2065501
rs9401616
rs4716795
rs7784347
rs1950861
rs4968174
rs111370608
rs73379542
rs7925466
rs6858961
rs62465767
rs7426276
rs78206502
All − MR Egger
All − Inverse variance weighted
−2
−1
0
1
2
MR effect size for
'Gut bacterial pathway abundance (PWY.6891..thiazole.biosynthesis.II..Bacillus.) || id:ebi−a−GCST90027600' on 'Pyogenic arthritis || id
